# Understanding heavy metal contamination in the vicinity of Lake Urmia, NW Iran: Environmental and health Perspectives

**DOI:** 10.1016/j.heliyon.2024.e34198

**Published:** 2024-07-05

**Authors:** Saeed Hosseinpoor, Shiva Habibi, Amir Mohammadi

**Affiliations:** a Department of Environmental Health Engineering, School of Public Health, Urmia University of Medical Sciences, Urmia, Iran; b Social Determinants of Health Research Center, Clinical Research Institute, Urmia University of Medical Sciences, Urmia, Iran

**Keywords:** Lake Urmia, Health risk, Settled dust, Heavy metal

## Abstract

This study addresses the potential impact of Lake Urmia on heavy metals (HMs) concentrations in the air and soil of the northern region of Lake Urmia in North West of Iran, highlighting significant environmental and health implications. The results showed different concentration levels for Arsenic (As), Cadmium (Cd), Chromium (Cr), and Lead (Pb) in soil and settled dust particles near Lake Urmia, and their concentrations exceeded recommended thresholds for Cr and Pb in some areas. Spatial distribution analysis indicated that local factors significantly affect contamination patterns, emphasizing the need for targeted interventions.

The study employed enrichment factor (EF) assessment and potential ecological risk (PER) index to identify pollution sources and evaluate associated ecological risks. The results indicated moderate to severe pollution levels in specific regions, particularly for Pb and Cd. Health risk assessments suggest that non-carcinogenic risks are generally below hazardous levels; however, concerns remain for Cr and As exposure.

Future studies should focus on long-term trends, source apportionment methodologies, and health effects of heavy metal exposure to develop effective pollution management strategies. Collaborative, interdisciplinary approaches will be crucial in mitigating heavy metal pollution and protecting human and environmental health.

## Highlights

• There is a growing concern regarding heavy metals contamination in the air and soil of Lake Urmia.
• Different concentration levels of arsenic, cadmium, chromium, and lead are reported near Lake Urmia.
• Analyzing enrichment factors and ecological risk indices helps accurately identify sources and assess risks.
• Prioritize long-term trend analysis, source identification, and health impact evaluation for effective pollution management.

## 1 Introduction

In recent decades, the concern about pollution, specifically the contamination of both land and air, has gained considerable attention due to heavy metals (HMs) accumulation in various environmental matrices [1]. HMs have different sources, stemming from activities such as fuel combustion, industrial processes, mining, and erosion of building surfaces [2]. Settled dust, which includes particles typically larger than 10 μm, plays a crucial role in the transport and deposition of these toxic elements onto the Earth's surface [3]. As an indicator of air pollution, settled dust has to be monitored and assessed due to its capacity to harbor HMs contaminants and its contribution to environmental and health impacts [4,5].

HMs, when present in the atmosphere and soil, pose significant health risks to humans [6]. These risks arise from toxic, non-biodegradable, and non-thermo-degradable nature of HMs, making them harmful to various organs, including the kidneys, liver, and bones upon exposure through ingestion, inhalation, or dermal contact [[7], [8], [9]]. Several studies worldwide have highlighted the ecological and health risks associated with HMs contamination in environmental media, including settled dust and street dust. The findings a research conducted across various urban functional zones in Shijiazhuang, China, revealed a significant enrichment in levels of Copper (Cu), Zinc (Zn), Cadmium (Cd), and Lead (Pb) in the dust. There was also substantial concentration variations between distinct functional areas, pointing to their anthropogenic sources [10]. A study on HMs pollution in Warsaw's street dust revealed a significant dependence on background values for calculating pollution indicators. Traffic-related pollution detection was more accurate with calculated indicators, while naturally elevated HMs concentrations led to underestimations. Aligning low concentrations with geogenic material better reflected pollution levels from moving vehicles [11]. Furthermore, investigations across six cities in Ebinur Lake Basin, China, investigated the spatiotemporal distribution of HMs in dust fall during heating and non-heating periods. Results revealed higher HMs levels in non-heating period, except for Cd and Pb, which were the highest during heating period. Main sources identified were fuel combustion, soil and industrial dust, and traffic emissions [12]. In research conducted in the vicinity of the former Aral Sea in the Republic of Uzbekistan, long-term exposure to saline dust and dust storms resulted in elevated levels of pollutant residues, and increased rates of illness and death, particularly for individuals with chronic diseases [13]. The investigation into dust storms and deposition in Aral Sea region emphasized the pronounced impact on the southern area due to prevailing wind direction. Findings from monitoring seven sampling stations between 2003 and 2012 indicated a generally low average dust deposition, yet notable peaks during dust storms, exhibiting seasonal patterns with heightened health-related threshold exceedance in spring and summer. The composition of dust samples closely resembled that of near-surface soils, and concentrations of HMs were relatively low [14].

In Baghdad, Iraq, Cd, Chromium (Cr), Zn, and Cu were found in dust samples, with implications for geoaccumulation and relative bioaccumulation [15].

Considering the multitude sources contributing to settled dust and its potential role in the dispersion of toxic elements, understanding the origins of HMs emissions in settled dust becomes paramount. The dearth of temporal trend surveys and the presence of high HMs concentrations in settled dust necessitate comprehensive evaluation of multi-source emissions and the associated health effects on both humans and ecosystem [16,17].

The northwestern region of Iran, surrounding Lake Urmia (saltwater lake), faces environmental challenges due to a combination of factors, including agriculture, industrial activities, and drying of over 90 % of the lake bed, primarily caused by climate change [18,19]. This region, covering an area of 80,000 km^2^ and home to more than 8,000,000 people by 2021, is vulnerable to environmental contamination [20].

In the northern regions of Lake Urmia, there are cities and villages with a total population of over 3,000,000. However, despite the crisis of Lake Urmia drying up, one of the world's largest saltwater lakes that is currently shrinking, comprehensive studies have not been conducted in this area. Identifying critical areas and concentrations of HMs is important, especially As, which has been identified with high levels in studies in other parts of the lake. However, the presence of HMs in the air and soil particles in this area poses a threat to the health of the residents.

Despite the threats to both the environment and public health, there has been limited research addressing HMs contamination in soil and settled dust within the northern regions of the former Lake Urmia. Hence, this study endeavors to assess HMs (As, Cd, Cr, and Pb) levels in surface soil and settled dust near the northern vicinity of Lake Urmia. Previous research had highlighted elevated levels of As, Cd, Cr, and Pb in another section of the Lake Urmia area. Additionally, these metals have significant health implications, as evidenced by previous studies [16,17,21]. Furthermore, a comprehensive examination of carcinogenic and non-carcinogenic risks linked to HMs exposure via inhalation, skin contact, and ingestion routes was conducted, employing advanced Monte Carlo simulation techniques.

## 2 Materials and methods

### 2.1 Study area and sampling

**Fig. 1:**
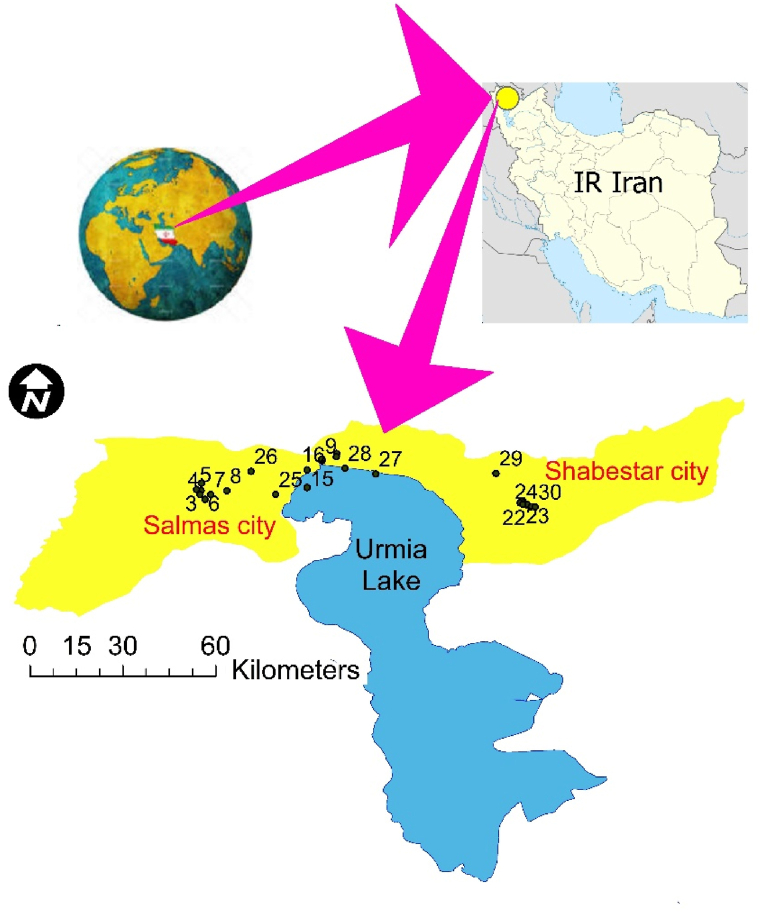
The study area map featuring sampling points, Shabestar city, Salmas cities, and the dried-up portion of Lake Urmia, exceeding 90 % of its original extent.

This study was carried out in the northern regions of the former Lake Urmia in the northwestern of Iran., specifically spanning from Salmas city to Shabestar city at coordinates 38° 22ʹ N, 44° 44ʹ E (Fig. 1). Meteorological data indicates that the area is primarily affected by humid winds from Atlantic and Mediterranean, resulting in chilly north winds and considerable winter snowfall. The mean annual temperature in this area is 12.6 °C, with temperature fluctuations ranging from −6 to 30 °C [22].

In March 2023, 30 samples of soil were gathered from this region, with three samples collected from each distinct area. The strategic sampling method was scheduled one week following the rainfall periods to minimize the potential impact of soil leaching and drainage on the concentrations of As, Cd, Cr, and Pb. Soil samples (0–30 cm) included collecting approximately 1 kg of soil (dry weight) as a composite sample from three subsamples situated approximately 10 m apart. Polyethylene bags were employed for sample collection and transportation to the laboratory for subsequent analyses.

Dust collection (30 samples) was done passively over a one-month period using a custom-designed sampler. The sampler comprised a standard electron microscope (SEM) stub base with a polycarbonate filter bed under a mesh protective cap featuring openings of 160 μm in diameter. It has two interconnected circular plates set 16 mm apart to facilitate air flow. A polycarbonate absorbent particle filter was affixed to the lower plate, providing protection against wind and rain. It was positioned at a height of 2 m from the ground at least 15 m away from any pollution source. The sampler collected particles through the combined forces of gravity, diffusion, and inertia. Zist Sepehr Bayhaq Company (accessible at https://zsbcompany.ir) is the creator of this sampler. After installation, the samplers were left in place for three months before being retrieved and transported to the analytical laboratory for further processing.

### 2.2 Sample preparation and chemical analysis

Preparation and digestion of settled dust samples adhered to the standardized procedure outlined in USEPA [[23], [24], [25]]. Initially, a Teflon beaker was employed for positioning the filter, followed by the addition of 10 mL of concentrated Suprapour nitric acid. The mixture was heated for 120 min on a hot plate at a temperature of 95 ± 5 °C until approximately 5 mL of the solution evaporated. Subsequently, 2 mL of ultrapure and 3 mL of H_2_O_2_ (30 %) were introduced. The heating process continued until ebullition ceased. Additional aliquots of 1 mL H_2_O_2_ (30 %) were added until no further change in the solution appearance was observed, the specified temperature was maintained for over 120 min until the solution volume reached 5 mL. In the final stage, 10 mL of concentrated hydrochloric acid was introduced into the solution and heated for 15 min.

The soil samples were then subjected to dehydration at 60 °C for 24 h, subsequently, the material underwent crushing and sieving through a 100-mesh (150 μm) sieve to simplify the determination of metal concentrations. The detection of HMs was conducted through the application of USEPA Method 3050B, as outlined in our earlier investigation [[23], [24], [25]].

HMs concentrations (specifically As, Cd, Cr, and Pb) were measured using ICP-AES, Arcus model, Germany. The method detection limit (MDL) was determined utilizing the standard deviation. The analytical method included examining samples demonstrating comparable retrieval rates to those from the field, achieving a recovery rate ranging between 83 and 96 percent of the original materials. Also, some of the analytical parameters for the ICP-AES were included a plasma gas flow rate of 15 L/min, an auxiliary gas flow rate of 1.5 L/min, a nebulizer gas flow rate of 0.7 L/min, RF power set to 1.2 kW, a wavelength range of 180–300 nm, a sample uptake delay of 15 s, and a sample uptake rate of 1 mL/min.

### 2.3 Quality assurance and quality control

Quality control measures included running blank digest solutions and conducting periodic rechecks of calibration standards accuracy and precision after every 20 samples. Calibration curve quality control was based on verifying the coefficient of determination (r^2^) in linear regression, with values exceeding 0.994 deemed acceptable. The MDL was calculated according to the USEPA-40 CFR part 136 recommendations, ensuring that the element value exceeded the predicted detection limit by 1–5 times and multiple measurements were taken.

This process entailed assigning the element value within a range of 1–5 times the estimated detection limit and performing multiple measurements. Following the calculation of the standard deviation, the MDL was determined using Formula 1:

(1) MDL=Student’stvalue×standarddeviation

The detection limits for As, Cd, Cr, and Lead (Pb) were 0.5 ppm, 0.1 ppm, 1 ppm, and 1 ppm, respectively.

### 2.4 Enrichment factors (EFs)

EFs were employed to ascertain potential sources of HMs and assess the extent of contamination. The EF value was calculated using formula (2):

(2) $$EF=\frac{{\left(C_{n}/C_{Fe}\right)}_{sample}}{{\left(C_{n}/C_{Fe}\right)}_{background}}$$

In this equation, C_Fe_ denotes the iron concentration, and C_n_ represents the quantity of HMs (mg/kg) in both the sampled site and the reference site. The contamination level of the samples was categorized based on EF values as follows: EF > 50 (very intense enrichment), 25 ≤ EF < 50 (very intense enrichment), 10 ≤ EF < 25 (extreme enrichment), 5 ≤ EF < 10 (relatively intense enrichment), 3 ≤ EF < 5 (moderate enrichment), 1 ≤ EF < 3 (slight enrichment), and EF < 1 (no enrichment).

### 2.5 Potential ecological risk (PER) and health risk assessment

In this study, the PER was employed to assess the ecological risks associated with the targeted metals.

The PER was calculated using (3), (4), (5):

(3) $$PER=\sum_{i=1}^{n}E_{j}^{i}$$

(4) $$E_{j}^{i}=T_{n}^{i}{\times \text{CF}}_{j}^{i}$$

(5) Cfi=CiCni

Here, CF represents the pollution factor specific to each metal, $C_{f}^{i}$ denotes the concentration of the target metal in the sample, and Cn is the background concentration of the target metal. Additionally, $E_{j}^{i\;}$ stands for the ecological risk potential factor of each metal, T represents the toxicity response factor of each metal, and PER indicates the overall ecological risk potential, which is derived from the sum of the PER of all investigated metals. The details regarding the PER were outlined in our earlier investigation [26].

The classification of PER entails assessing the ecological risk potential factors for individual metals ($E_{j}^{i\;}$) and the overall PER. Each metal risk potential range ($E_{j}^{i\;}$) is segmented into various risk degrees. Furthermore, the PER as a whole is categorized into corresponding risk degree classifications. Metals exhibiting a risk potential below 40 are categorized as posing low risk. Those with risk potentials falling within the range of 40–80 are regarded as having moderate risk potential. Metals with risk potentials ranging from 80 to 160 are designated as being subject to investigation. The PER is considered high for metals ranging from 160 to 320, and anything surpassing 320 is labeled as severe. Similarly, the classifications for the overall PER are determined by evaluating the cumulative risk potential.

#### 2.5.1 Exposure assessment

To assess human exposure to HMs present in surface soil dust, three exposure pathways were considered including ingestion, inhalation, and dermal absorption. The average daily doses (ADDs) for each pathway (ADDing, ADDinh, ADDderm) in mg/kg.day were calculated using the USEPA method [27]:

(6) $$ADD_{ing}=\frac{Csoil\times IngR\times EF\times ED}{BW\times AT}\times CF$$

(7) $$ADD_{inh}=\frac{Csoil\times InhR\times EF\times ED}{PEF\times BW\times AT}$$

(8) $$ADD_{derm}=\frac{Csoil\times SA\times AFsoilABS\times EF\times ED}{BW\times AT}\times CF$$

The coefficients for these equations are detailed in our previous study [28]. Non-carcinogenic risk assessment involved computing the hazard quotient (HQ) for individual elements and the hazard index (HI) representing the cumulative HQ across three exposure pathways.

(9) HQ=ADD/Rf

(10) $$HI=\sum HQ=HQ_{ing}+HQ_{inh}+HQ_{derm}$$

HQ or HI values exceeding 1 suggest the possibility of non-carcinogenic effects, whereas HQ or HI values below 1 indicate a safe threshold for future exposure.

The calculation of the cancer risk linked to prospective exposure involved determining the excess lifetime cancer risk (ELCR) using Formula 11.

(11) ELCR=ADD×CSF

(12) $$\sum \text{ELCR}=ELCR_{ing}+ELCR_{inh}+ELCR_{derm}$$

Based on the ELCR association, the Cancer Slope Factor (CSF) serves as the cancer quality factor, with its specific values referenced from relevant sources for the involved variables. Total ELCR values ranging from 10^-6 to 10^-4 suggest an acceptable or manageable risk. Values below 10^-6 are considered negligible, while those exceeding 10^-4 indicate a significant cancer risk for humans [27].

#### 2.5.2 Monte Carlo simulations and sensitivity analysis

A Monte Carlo simulation method using Crystal Ball software (version 11.1.2.4, Oracle, Inc., USA) was employed for sensitivity analysis, involving 1000 repetitions. This stochastic model aims to mitigate uncertainty in risk estimation compared to deterministic methods that rely on single-point variables. The advantage of this method lies in its ability to demonstrate the impact of each parameter on the estimated risk values; parameters with higher coefficients exert more significant effects [29].

### 2.6 Statistical analysis

Statistical treatment of data collected at sampling stations and their interrelationships involved the use of descriptive statistics, encompassing metrics like minimum, maximum, percentile, median, mean, standard deviation, and correlation analysis using Stata 14 software. Additionally, spatial analysis was conducted using ArcGIS 10.1, employing the kriging interpolation method to generate independent raster layers, which were then visualized and analyzed.

## 3 Result and discussion

### 3.1 Heavy metal levels

Table 1 reveals that the mean concentrations of As, Cd, Cr, and Pb in soil samples were 10, 0.24, 84, and 22 mg/kg, respectively. The ranking of HMs was determined as Cr > Pb > As > Cd. Additionally, the mean concentrations of As, Cd, Cr, and Pb in settled dust particle samples were 9, 4, 6, and 2 mg/kg, respectively. The ranking of HMs in settled dust particles was determined as As > Cr > Cd > Pb. The concentrations of HMs in settled dust particles were lower than the recommended values for environmental protection agency in Iran. However, in soil samples, 23 % of the sampling sites exceeded the recommended range for Cr, 6 % for Pb, and 3 % for As. Table 2 shows HMs levels compared to the other areas of the world. According to the results, As levels were lower than those observed in studies from Canada [30], Mongolia [31], and Kerman (Iran) [32]. Likewise, the levels of Cd, Cr, and Pb were below those reported in studies from Canada and Germany [33]. However, Cd levels in settled dust were higher, and Mongolian studies indicated even greater Cd levels in settled dust. This discrepancy may be related to road traffic and transportation activities.

**Table 1:** Statistical descriptive of HMs in soil (n: 30) and settled dust (n: 30) samples in the study area (μg/L).

Element	Soil (n: 30)					Settled dust (n: 30)				
	As	Cd	Cr	Pb	Fe	As	Cd	Cr	Pb	Fe
Mean	10	0.24	83	22	2.9 %	9	4	6	2.3	3 %
Std. deviation	7	0.03	47	41	0.4 %	2	1	1	0.4	0.3 %
Median	10	0.24	67	11	2.9 %	10	4	6	2.3	2.9 %
Max	44	0.29	220	211	3.8 %	13	6	9	3.2	3.8 %
Min	2.50	0.15	37	6	2.1 %	5	3	4	1.7	2.2 %
CV	0.72	0.13	0.57	1.85	0.14	0.24	0.23	0.19	0.18	0.12

**Table 2:** Heavy metal contents in some areas of the world and comparison with soil quality recommendations (mg/kg).

Element	As	Cd	Cr	Pb	Fe	Type	Source
Mean	10	0.24	83	22	2.9 %	Soil	**This study**
	9	4	6	2.3	3 %	Settled dust	
Background soil	4.1	0.2	43	6	% 2.9	soil	
Dried bed Urmia Lake	12.1	0.5	38	10	1.8 %	Bed of Lake Urmia	
Background of Isfahan, Iran		0.26	85	28	2.6 %	soil	[34]
Background of Turkey	8	0.2	93	33	2.6 %	soil	[35]
Background of Germany	N.D	1.50	45	171		soil	[33]
Iran-EPA guidelines	18	1	110	50	–	Agricultural land	[36]
Canadian soil	12	1.4	64	70		Agricultural land	[30]
Ulaanbaatar, Mongolia	16	4.8	70	51	–	Settled dust	[31]
Kerman, Iran	10.9	0.34	28	45		Road Dust	[37]
Earth's soil	5	0.11	70	29	3.2 %		[32]
Earth's crust	1	0.6	0.1	14	4.1 %		[32]

The concentrations of As, Pb, and Cr in settled dust particles in the northern region were lower than those in the surrounding areas of the lake. It could be due to the presence of water in the lake bed near the northern regions, as previous reports indicate that areas extending to the west and east of the lake had higher concentrations due to the dryness of the lake bed in those areas [38].

The present study computed low coefficient of variance (CV) values (CV < 1) for all soil sampling sites in relation to HMs except for Pb. The highest CV was observed in soil samples, particularly for Pb (1.85), As (0.72), Cr (0.57), and Cd (0.13). This indicates a much greater variability in Pb concentrations across the sampling sites, which can skew the overall analysis and interpretation of HMs distribution. The variability of HMs in both soil and settled dust samples can be assessed using CV. For settled dust samples, all CV values were under 0.5, with As having the highest CV of 0.24. The CV values falling within the range of 21 %–50 % indicate moderate variability, while those between 50 % and 100 % suggest a higher degree of variability, and CV values surpassing 100 % indicate severe variability [39]. A study conducted in four cities (Balkhash, Ust-Kamenogorsk, Ridder, and Shymkent) in Kazakhstan aimed to determine soil contamination levels. The CV for metals such as Pb, Cd, Cu, Zn, and Cr ranged from 35.4 to 282.3 % across all cities. Kazakhstan, due to the consequences of the drying up of Aral Sea and anthropogenic activities, is very similar to arid studies [40]. Research undertaken in Hangzhou, China, revealed that soil concentrations of As, Cd, Cr, and Pb met environmental standards, yet As and Cd surpassed background levels due to human influence. The CV for these metals was below 1. As and Cd exhibited higher concentrations with a CV of 0.5 < CV < 1, suggesting non-uniformity. These outcomes were in line with those of the present investigation although some metal origins in our study were linked to the desiccated lake bed, contributing to soil and air pollution through dust dispersion [41].

A distinct study focusing on sediments from the Vistula River and surrounding soils in Poland, situated in the heart of Europe, found elevated levels of HMs in the sediments relative to the soil. This underscores the significance of addressing the wider ramifications of climate change, which go beyond immediate issues like droughts and water scarcity, potentially affecting extensive environmental regions and jeopardizing biodiversity. The ongoing drought trend in the Middle East, akin to the outcomes of this research, implies similar repercussions for drying lakes like Lake Urmia and the Aral Sea [42].

### 3.2 Spatial distribution and source apportionment of HMs in soil and settled dust samples

According to USEPA recommendations, the common method for determining the sources of metal emissions in soil and air sinks is principal component analysis (PCA). In this study, the results of PCA analysis are shown in Fig. S1 and details are presented in Table 3. Three main groups were obtained for soil samples and sedimented dust particles, including the first group of As and Pb, the second group of Cd, and the third group of Cr. Generally, for Pb and As, vehicular traffic and the lakebed substrate as Aeolian dust are potential sources; for Cd, agricultural activities; and for Cr, natural background and soil origins of the region may be responsible. These origins are mentioned in other studies [[43], [44], [45], [46]].

**Table 3:** Principal component analysis of heavy metal in soil and atmospheric settled dust.

Variable	Component in soil			Component in settled dust		
	1	2	3	1	2	3
As	**0.62**	−0.13	0.11	**0.55**	−0.06	−0.62
Cd	0.22	**0.93**	0.27	0.46	−0.49	**0.68**
Cr	−0.42	0.14	**0.86**	0.35	**0.86**	0.33
Pb	**0.58**	0.30	0.39	**0.59**	−0.05	0.16
Eigenvalue	2.09	0.96	0.79	2.2	0.87	0.56
Variance%	52	24	19	56	21	11
Cumulative variances%	52	76	96	56	78	92

**Fig. 2:**
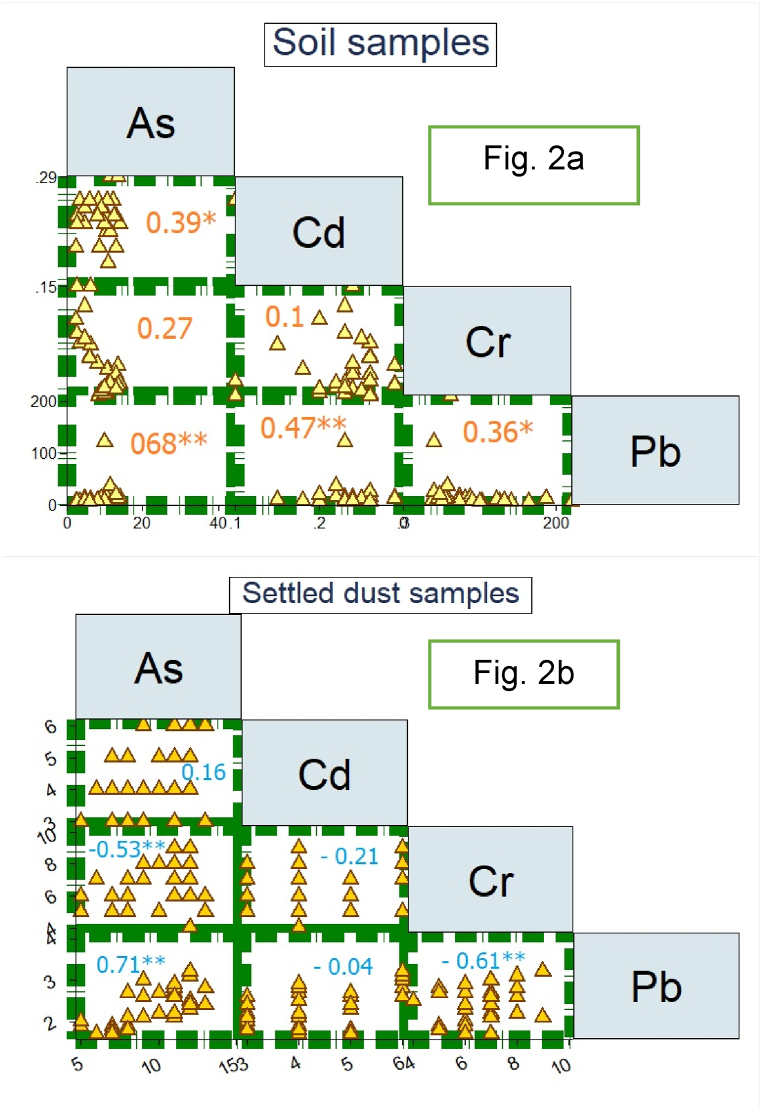
Spearman correlation coefficients between HMs values in soil (Fig. 2a) and settled dust (Fig. 2b) samples (*P_value_<0.05, ** P_value_<0.01).

The correlation between HMs is another method for identifying emission sources. The Spearman correlation matrix in Fig. 2 (a and b) illustrates the relationships among arsenic As, Cd, Cr, and Pb. In soil and settled dust samples, the distribution of HMs was irregular due to weak correlations, except for a relatively strong correlation between As and Pb. Overall, significant correlations between HMs in soil and settled dust particles were not found (R > 0.9, P-value <0.05). However, a correlation between As and Pb in both soil and air particles sinks indicated similar emission sources. These findings were confirmed by PCA [41].

In order to delve deeper into the spatial variances of metals and determine their origins, we employed Ordinary Kriging. This technique facilitated the representation of these variances through the generation of color-coded raster maps. Rooted in a stochastic framework, this method highlights regions with elevated concentrations using vivid red markings, while regions with lower concentrations are depicted with green hues. The map illustrating the zoning of HMs in the soil is presented in Fig. S2. These maps exhibit analogous dispersion patterns, with the eastern portion of the study area indicating areas of heightened activity. Different factors contribute to soil contamination including dust, the arid lakebed substrate, agricultural practices, vehicular traffic, and industrial activities. Spatial analysis revealed different dispersion patterns for Cd and Cr settled dust (Fig. S2). This disparity can be attributed to their distinct emission sources, while Pb and As displayed congruent distribution patterns in settled dust. The sediment substrate of the lake may have an effect in this regard [17,38]. Hotspots for Pb and As were identified in the eastern sectors of the study area, notably in Shabestar city. An essential consideration regarding pollution distribution is the predominant wind direction, which blows from west to east in this vicinity and potentially carries pollutants towards Shabestar. Previous studies have cited pesticides and chemical fertilizers as sources of Cd. The source of Cr in this study may originate from natural sources such as lithogenic components and soil parent materials, consistent with prior studies [[47], [48], [49]]. However, As and Pb likely stem from transit routes and the dry lakebed substrate [17,38].

### 3.3 EFs assessment

**Fig. 3:**
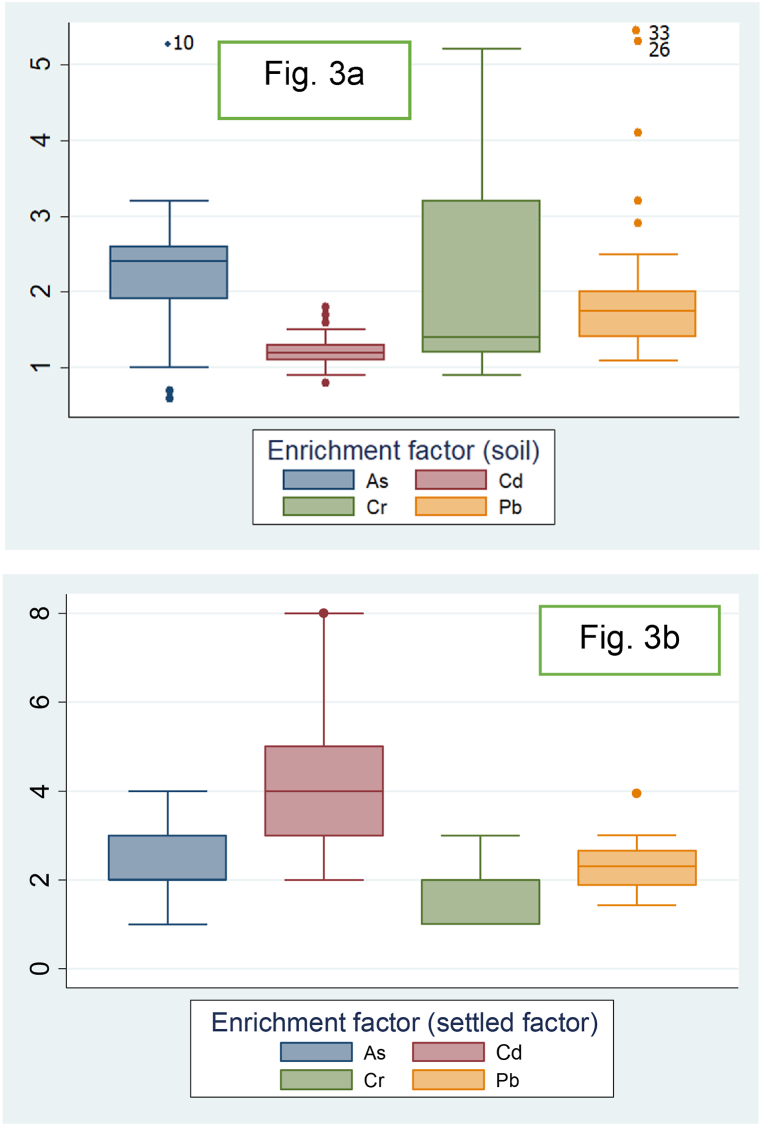
Descriptive statistic of enrichment factor values in soil (Fig. 3a) and settled dust (Fig. 3b) samples.

Exploring soil pollution often involves utilizing EFs as a method to pinpoint potential sources of HMs, whether originating from natural occurrences or external human activities. In Fig. 3, box plots illustrate the descriptive statistics of EF for four HMs.

The mean EF values for As, Cd, Cr and Pb were 2.3, 4.2, 1.6, and 3.8, respectively, in settled dust samples, and 2.49, 1.19, 1.94, and 3.73 in soil samples. As and Cr in settled dust samples were characterized as undergoing slight enrichment, while Cd (in 83 % of sampled areas) and Pb (in 76 % of sampled areas) were labeled as experiencing moderate enrichment. Soil samples display similar patterns; Pb (in 13 % of sampled areas), As (in 20 % of sampled areas), and Cr (in 16 % of sampled areas) fall under the moderate enrichment classification. Cd is categorized as experiencing slight enrichment. Additionally, human activities such as road traffic and dust from the dry lakebed could contribute to the increased EFs. These findings have been noted in previous studies conducted in Iran and other countries [[50], [51], [52]].

### 3.4 PER index

As a deterministic method for determining the sensitivity of nature and organisms to the toxicity of HMs, the PER is commonly used in soil and airborne particle studies [53,54]. The PER index ($E_{j}^{i\;}$) for As, Cd, Cr, and Pb, along with separate PER for soil and airborne particles, is presented in Table 4. The $E_{j}^{i\;}$ values, along with the toxicity classification in soil, were identified as Cd > As > Pb > Cr, and in settled dust particles as Cd > Pb > As > Cr.

**Table 4:** Ecological risk ($E_{j}^{i}$) of each HMs and index (n: 30).

Sampling zones (S.Z)	$E_{j}^{i}$								PER	
	Soil samples				Settled dust samples					
	As	Cd	Cr	Pb	As	Cd	Cr	Pb	Soil	Settled dust
Mean	25	36	4	19	2.3	13.0	0.3	12	83	27
St. deviation	18	5	2	34	0.6	3.1	0.1	2.2	50	5
Max	108	44	10	176	3.2	18.0	0.5	16	326	37
Min	6	23	2	5	1.2	9.0	0.2	9	49	20
$E_{j}^{i}$ <40	97 % (S.Z)	90 % (S.Z)	100 % (S.Z)	93 % (S.Z)	100 % (S.Z)	100 % (S.Z)	100 % (S.Z)	100 % (S.Z)		
40≤ $E_{j}^{i\;}$ <80		10 % (S.Z)								
80≤ $E_{j}^{i\;}$ <160	3 % (S.Z)			7 % (S.Z)						
PER <150									93 % (S.Z)	100 % (S.Z)
300≤ PER ≤600									7 % (S.Z)	

**Fig. 4:**
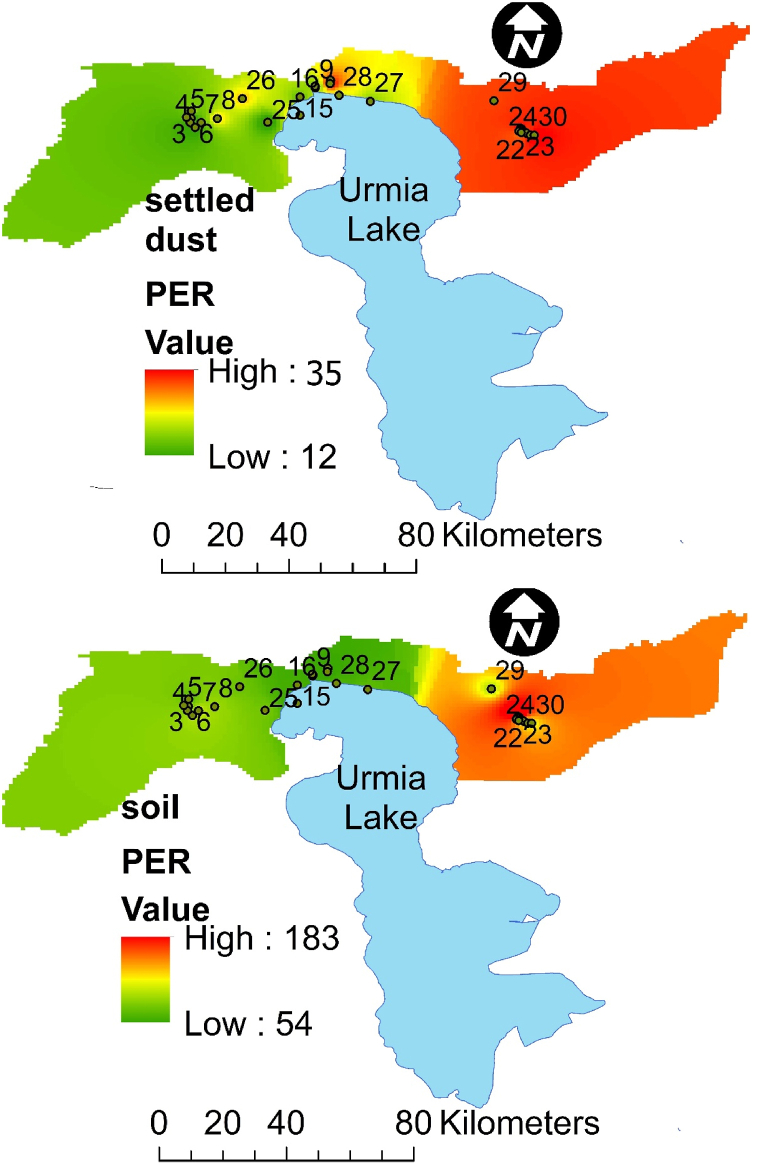
Spatial distribution of potential ecological risk in north area of Lake Urmi

The highest ecological risk index for Cd and Pb was observed in 7 % of the sampling areas, and considerable $E_{j}^{i\;}$ values were classified at considerable risk. These areas are aligned with the eastern regions of the study area, as indicated in the zoning map (Fig. 4). Airborne particle samples were classified as low-risk $E_{j}^{i\;}$. The $E_{j}^{i\;}$ levels for As, Cd, and Cr were categorized as "negligible" with low risk. Previous studies have reported high risk relative to Cd [52,55]. The total pollution index PER in 7 % of the soil sampling areas was identified as severely polluted. These areas are situated in the eastern region of the study area, as depicted in zoning map in Fig. 4. However, both central and eastern regions exhibited higher sensitivity to HMs in sedimented dust particles. Ultimately, based on the ecological risk index calculated for HMs in sampling areas, low risk (PER <150) was estimated in 93 % of the sampling locations.

### 3.5 Health risk assessment and sensitivity analysis

Table 5 illustrates the non-carcinogenic and lifetime carcinogenic risks associated with dermal, inhalation, and ingestion exposures based on deterministic modeling. The gastrointestinal route is the main exposure pathway to humans for HMs. Non-carcinogenic risks, with HQ < 1, were deemed non-hazardous in all areas. However, Pb and As had HQ values higher than Cd and Cr, warranting attention. These findings are consistent with previous studies [28,41].

**Table 5:** Carcinogenic and non-carcinogenic cancer risks.

Exposure source	Hazard quotient	As	Cd	Cru	Pb	Total metals
Soil	mean	8.86E-03	6.05E-05	2.47E-03	1.42E-03	1.26E-02
	SD	6.25E-03	7.93E-06	1.39E-03	2.62E-03	
Settled dust	mean	8.11E-04	1.10E-04	1.92E-05	1.49E-04	1.08E-03
	SD	1.96 E −04	2.61E-05	3.79E-06	2.82E-05	
**Cancer risk**		**Total pathways**				
Soil	mean	3.98E-06	3.77E-07	1.10E-05	4.88E-08	1.50E-05
	SD	2.94E-06	4.16E-08	6.20E-06	8.75E-08	
Settled dust	mean	3.77E-07	6.79E-07	8.55E-08	6.97E-09	1.15E-06
	SD	9.13E-08	1.61E-07	1.69E-08	1.11E-08	

In soil samples, the mean ELCR values for Cd, As, Cr, and Pb through all exposure pathways were within the acceptable risk range (<10^−5^). However, Cr in soil was higher than the other HMs. The average gastrointestinal ELCR for As, Cd, and Cr in soil was Cr > As > Cd > Pb, and in airborne particles was Cd > As > Cr > Pb. As and Cr posed significant risks, which is consistent with findings of studies conducted around the lake [28,56,37].

The cumulative carcinogenic risk of the four metals in soil and airborne particles was less than.

10^−3,^ which was higher than similar studies, indicating the need for further control and supplementary studies. Despite being transported through dermal, oral, and respiratory routes, As posed minimal risks [[57], [58], [59]].Mont Carlo simulation as a stochastic model (Fig. S4) confirms these results. Sensitivity analysis was utilized to determine the significant role of the most influential factor in potential carcinogenicity associated with HMs exposure. In this stochastic model, uncertainty has been incorporated to predict the ELCR, and the values of mean, standard deviation, 95th percentile, and 5th percentile are detailed in Fig. S4. Sensitivity analysis indicated that As concentration in soil and particles, Cd and Cr IngR, and Pb exposure duration in soil with exposure frequency in settled dust were the most important factors in carcinogenic risk. These results may vary due to the uncertainty between regions and day-to-day variability. The results showed an inverse correlation in exposure pathways and carcinogenicity. Previous studies have confirmed this finding [28,38].

#### 3.5.1 Strengths and weaknesses

The extensive collection and analysis of data on heavy metal concentrations in soil and settled dust samples, along with the use of advanced statistical methods such PCA and Ordinary Kriging for spatial distribution assessment, and the detailed health risk evaluation considering sensitivity analysis, provide a solid foundation for understanding the environmental and health effects of heavy metal contamination. However, there are weaknesses that need to be addressed. This study requires supplementary research. The variability in heavy metal distribution, particularly for Pb, needs more in-depth analysis to clarify data inconsistencies. Comparisons with recommended environmental standards and findings from other regions should be more detailed in supplementary studies to strengthen the study's context. Ensuring rigorous rechecking and validation of results against existing literature will be essential to confirm the accuracy and reliability of the conclusions in the future.

#### 3.5.2 Suggestions for future research

• Investigate the long-term trends of HMs concentrations in soil and settled dust particles to assess temporal variations and potential impacts of climate change.
• Conduct detailed source apportionment studies using advanced techniques such as receptor modeling to identify specific anthropogenic and natural sources of HMs pollution.
• Explore the potential health effects of chronic exposure to HMs in vulnerable populations, such as children and pregnant women, through epidemiological studies.
• Assess the effectiveness of current pollution control measures and remediation strategies in mitigating HMs contamination in soil and air sinks.
• Investigate the role of emerging pollutants and their interactions with HMs in influencing environmental and human health outcomes.
• Explore the impact of land-use changes and urbanization on HMs pollution patterns to inform sustainable land management practices.
• Investigate the fate and transport mechanisms of HMs in different environmental compartments, including soil, water, and biota, to better understand their environmental behavior.
• Conduct interdisciplinary studies integrating environmental science, public health, and social sciences to develop holistic approaches for addressing HMs pollution and its societal impacts.
• Explore innovative technologies and monitoring techniques for real-time detection and assessment of HMs pollution in soil and air sinks.
• Collaborate with stakeholders, including local communities, policymakers, and industry partners, to develop and implement integrated pollution management strategies aimed at reducing HMs exposure and protecting human and environmental health.

## 4 .Conclusion

This study provides valuable insights into the levels, distribution, and potential risks associated with heavy metal pollution in soil and settled dust particles in the study area. The findings reveal that Cr, Pb, As, and Cd are among the prevalent HMs, with varying concentrations observed in different environmental compartments. Notably, while settled dust particles generally exhibit lower HMs concentrations compared to soil samples, certain areas still exceed recommended limits, particularly for Cr, Pb, and As in soil.

The spatial distribution analysis highlights the influence of local environmental factors, such as proximity to water bodies, on HMs concentrations, emphasizing the need for comprehensive understanding and monitoring pollution sources. Moreover, the low CV observed in settled dust samples suggests relatively uniform contamination patterns, contrasting with the higher variability observed in soil samples.

Further investigation into the sources of HMs pollution, including anthropogenic activities and natural processes, is essential for developing targeted mitigation strategies. Advanced techniques such as PCA and ordinary kriging can aid in identifying emission sources and spatial variability, informing pollution control measures and land management practices.

Health risk assessments underscore the importance of considering exposure pathways and cumulative risks associated with HMs contamination. Although non-carcinogenic risks were generally deemed non-hazardous, attention is warranted for Cr and As due to their higher HQ values.

Finally, this study employs advanced techniques like PCA and ordinary kriging to provide innovative insights into the spatial distribution and sources of HMs pollution. By integrating comprehensive health risk assessments, it offers a novel understanding of contamination patterns in soil and settled dust particles. These findings inform targeted mitigation strategies and effective pollution management practices.

To address the gaps identified in this study and contribute to effective pollution management, future research endeavors should focus on long-term trends, source apportionment, health effects, and innovative monitoring technologies. Collaboration among stakeholders, interdisciplinary research approaches, and community engagement will be crucial in developing sustainable solutions to mitigate heavy metal pollution and safeguard human and environmental health.

## Data availability

All the relevant data are included in the manuscript and the supplementary document. No separate repository is attached.

## CRediT authorship contribution statement

**Saeed Hosseinpoor:** Methodology, Formal analysis. **Shiva Habibi:** Writing – original draft, Investigation. **Amir Mohammadi:** Writing – review & editing, Writing – original draft, Project administration.

## Declaration of competing interest

The authors declare that they have no known competing financial interests or personal relationships that could have appeared to influence the work reported in this paper.
