# Supplementary material for: Understanding heavy metal contamination in the vicinity of Lake Urmia, NW Iran: Environmental and health Perspectives
Source: Heliyon. 2024 Jul 5;10(13):e34198. doi: 10.1016/j.heliyon.2024.e34198 (PMC11277387; doi:10.1016/j.heliyon.2024.e34198)
Supplement: Multimedia component 1 [file mmc1.docx]

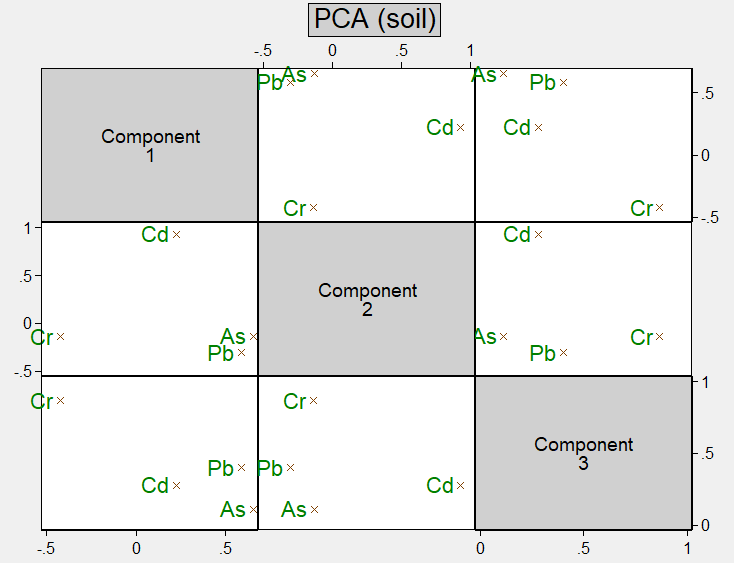


Figure S1 Biplot for the 3 principal components


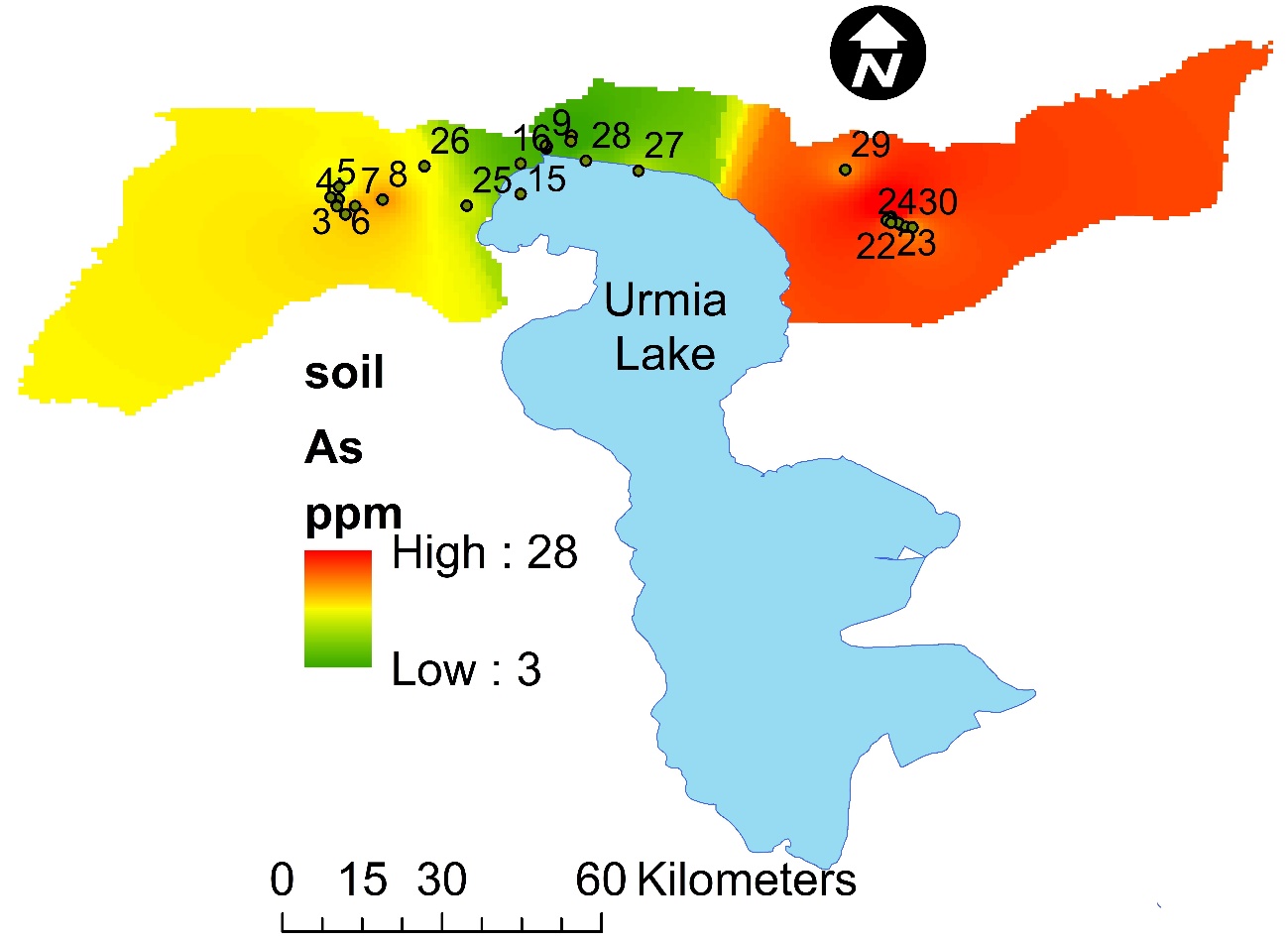

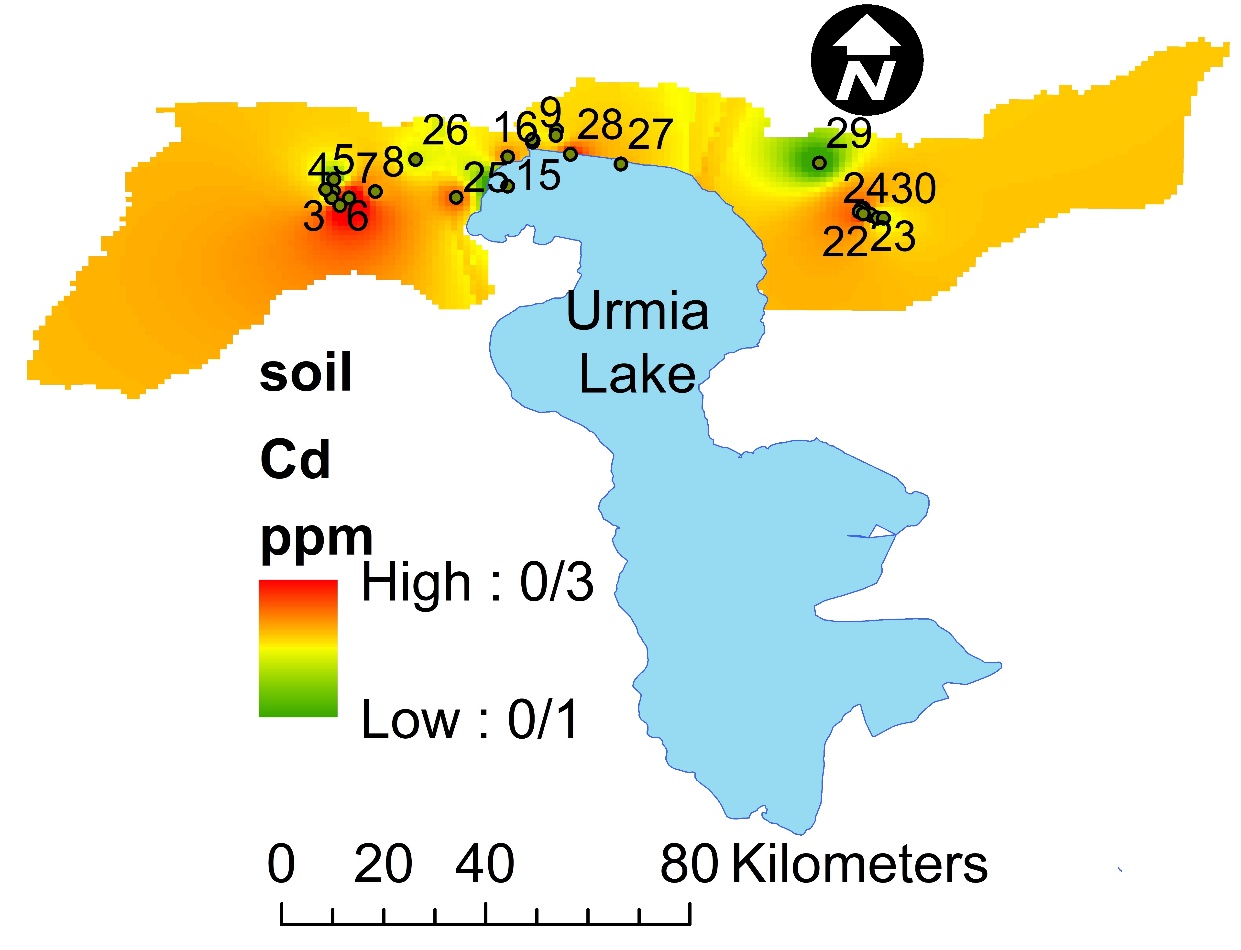

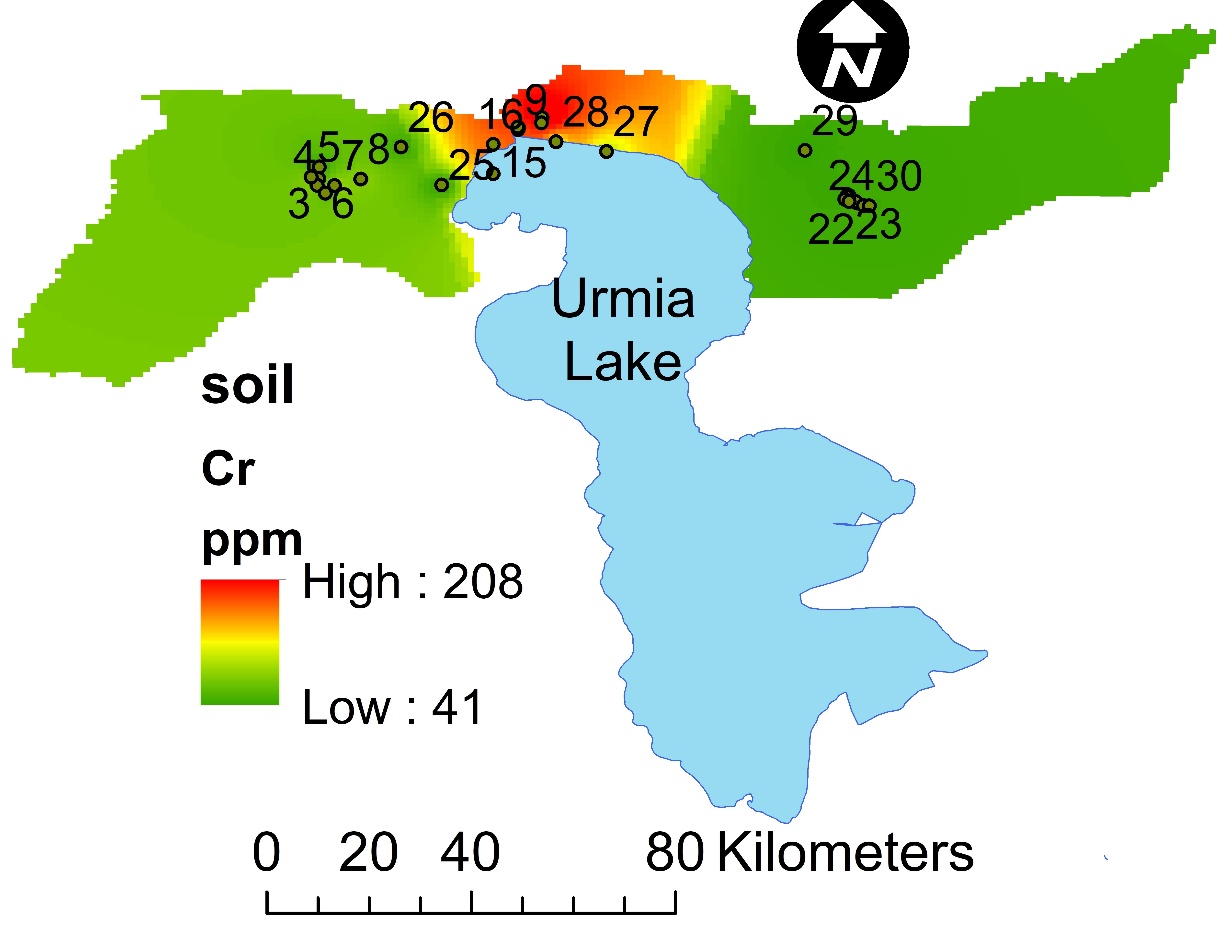

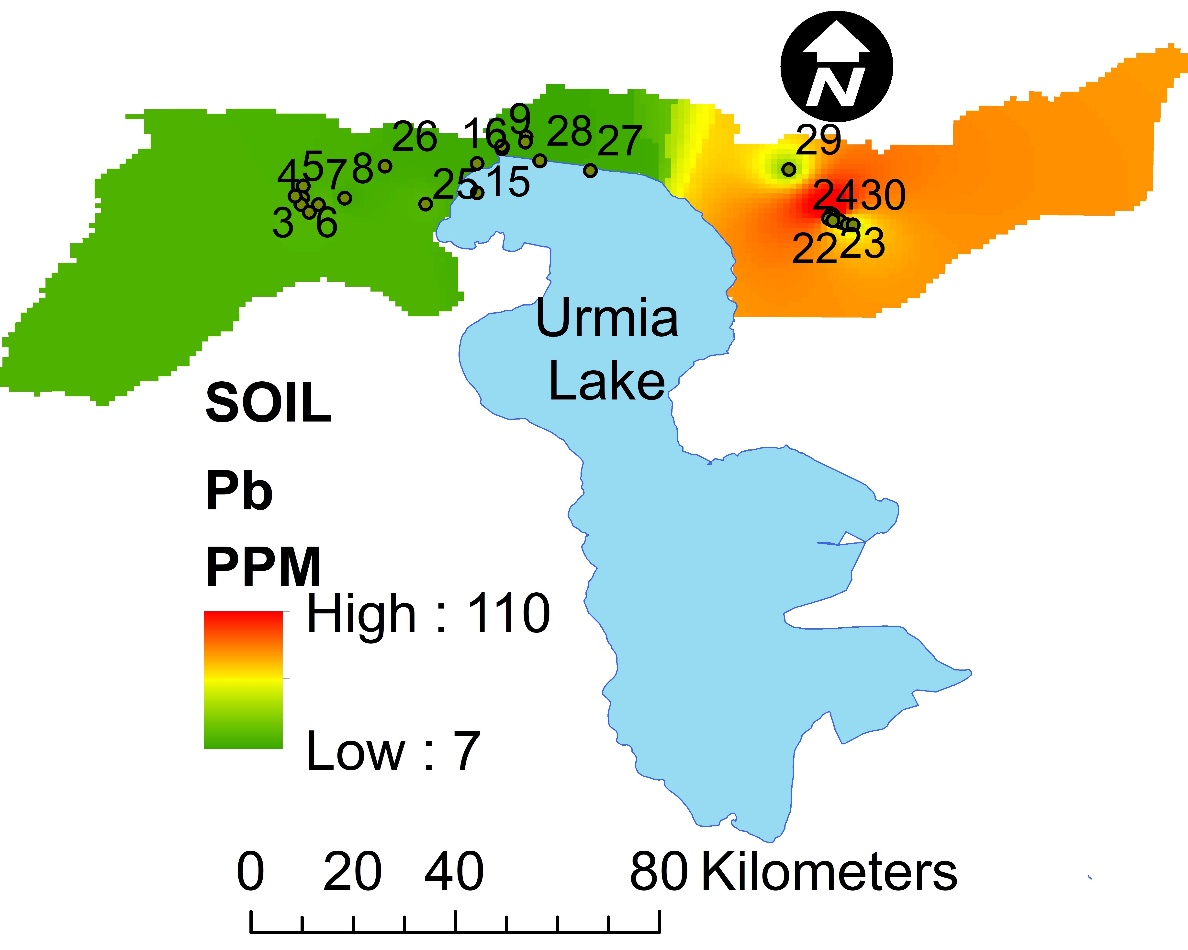


Figure S2 Spatial distribution of HMs in soil


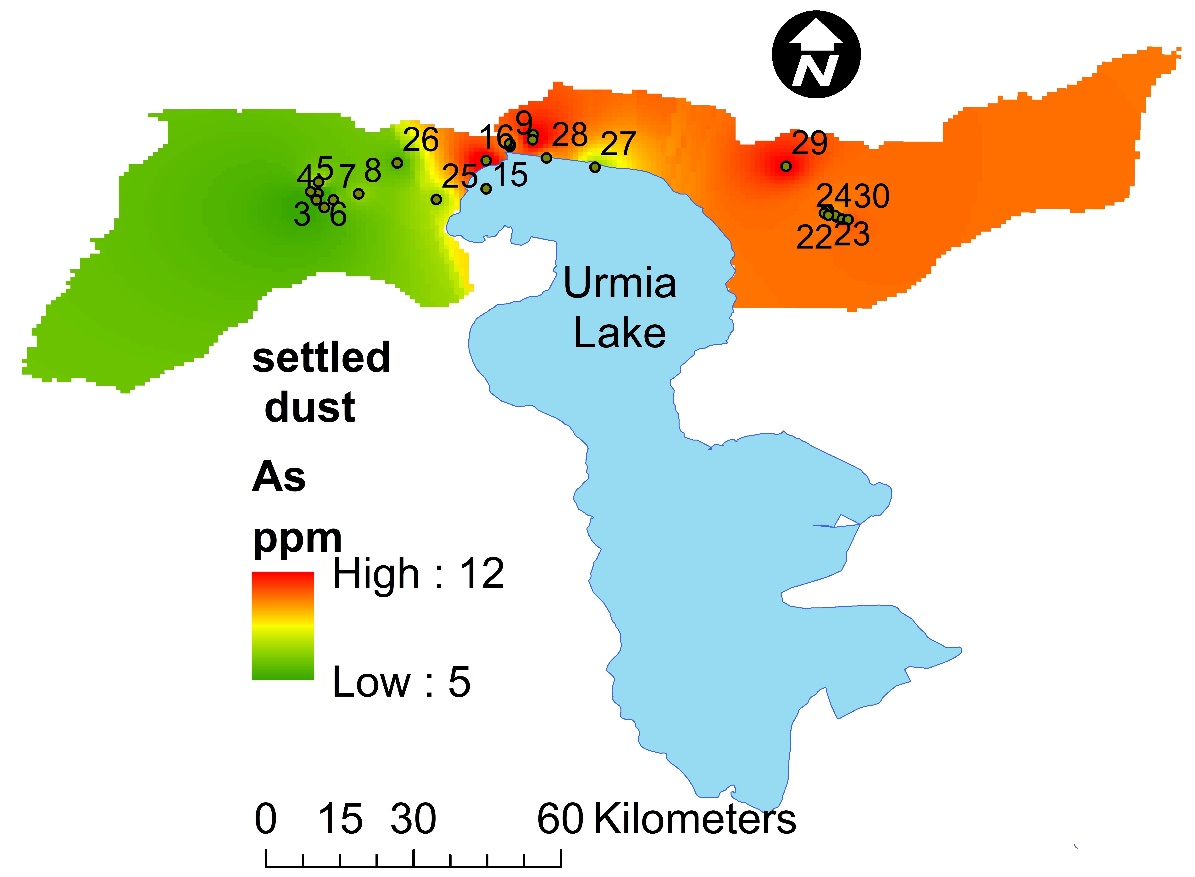

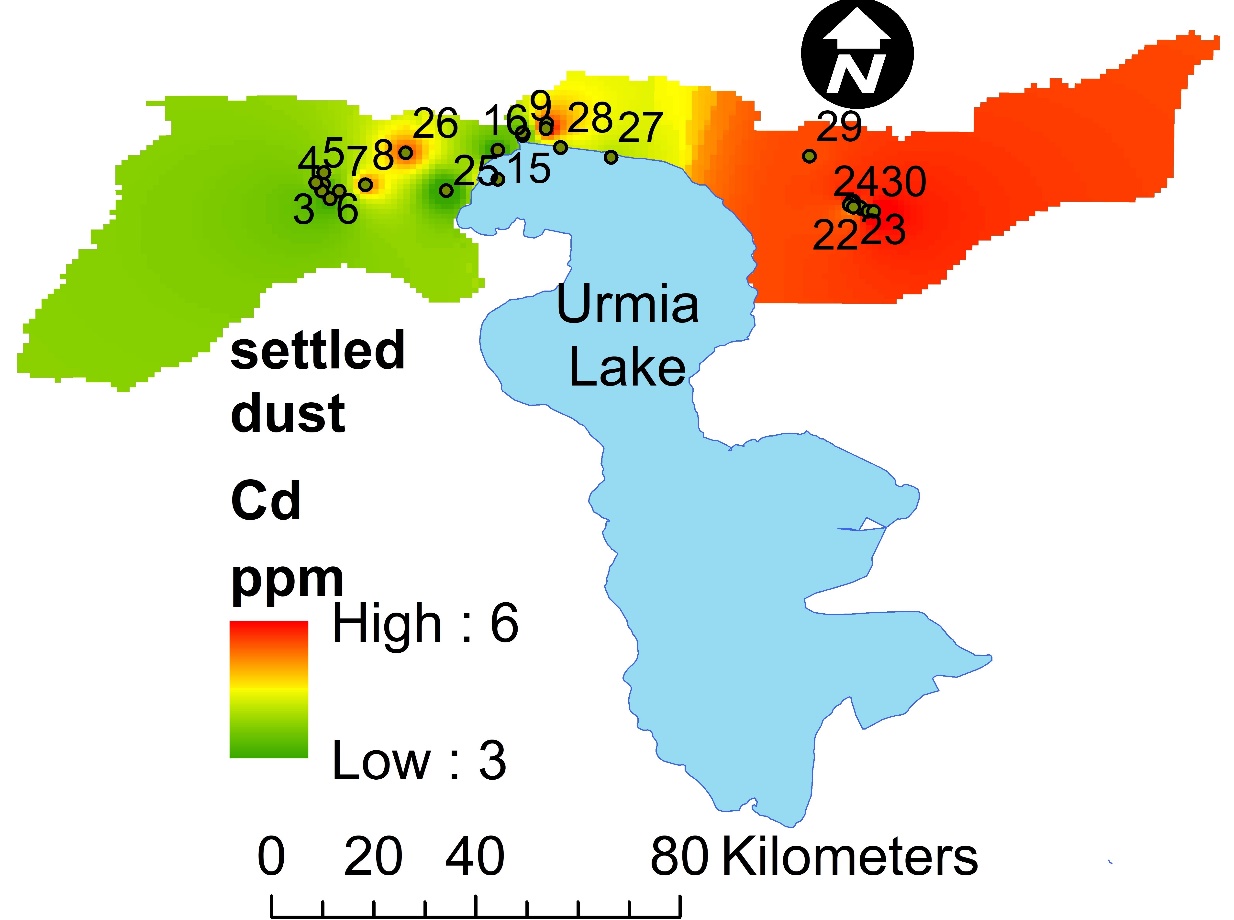


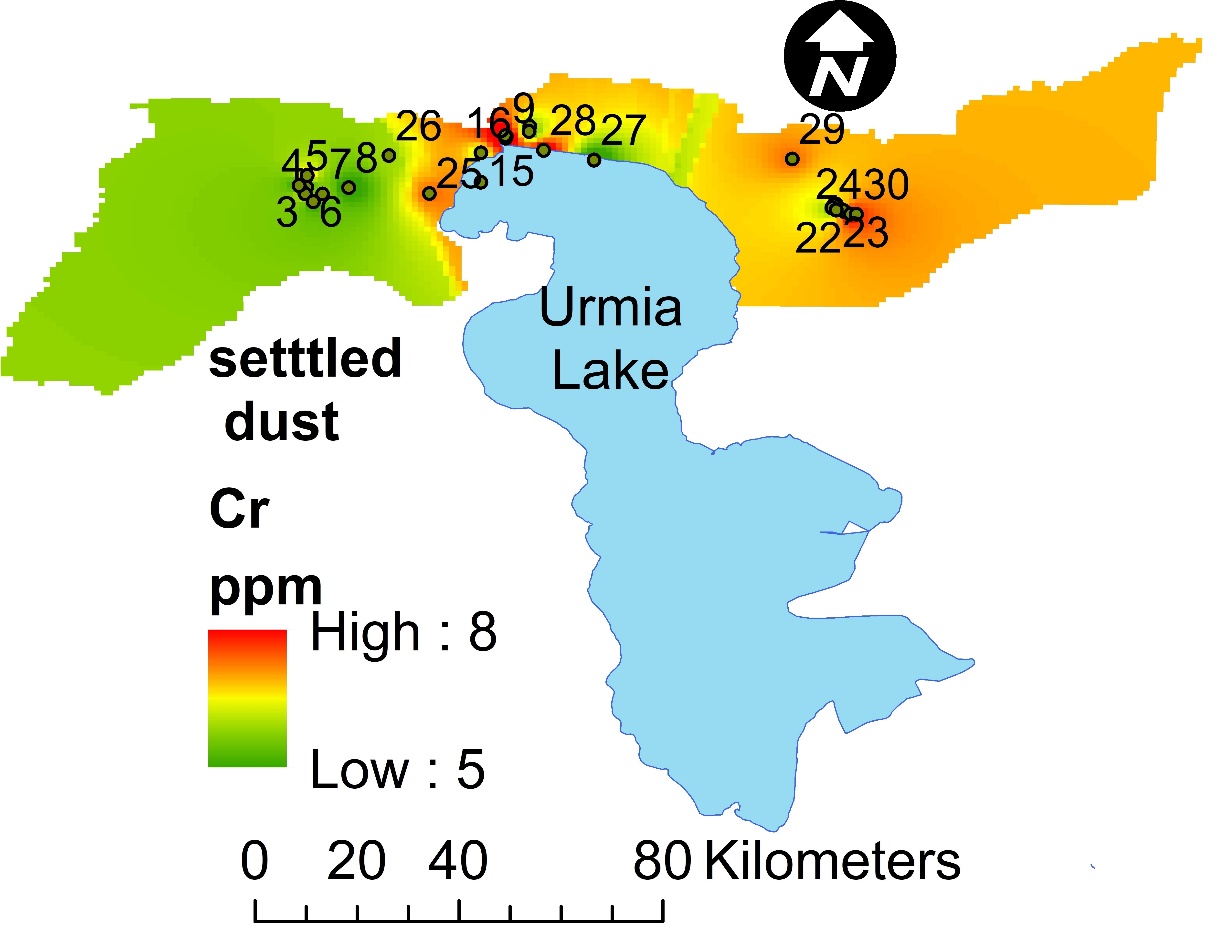

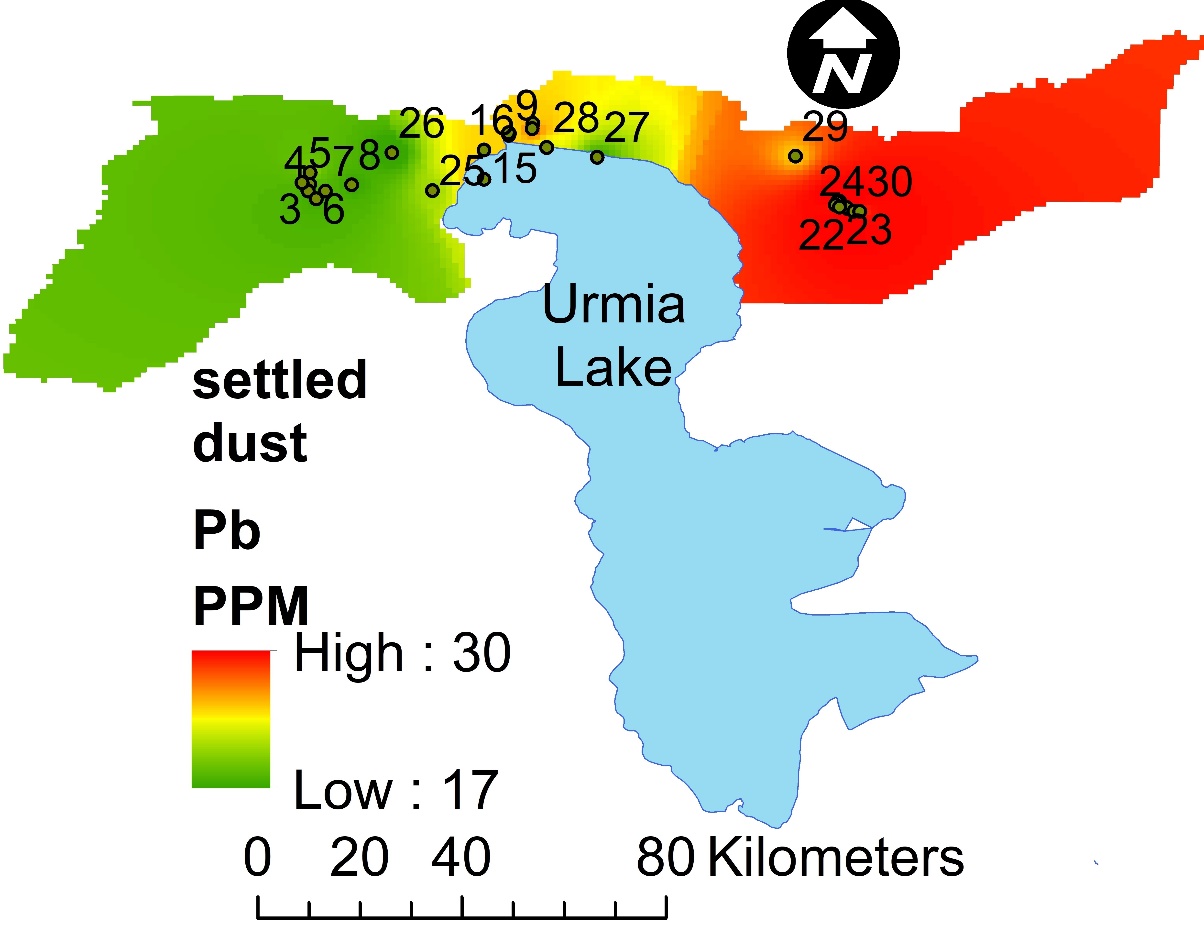


Figure S3 Spatial distribution of HMs in settled dust


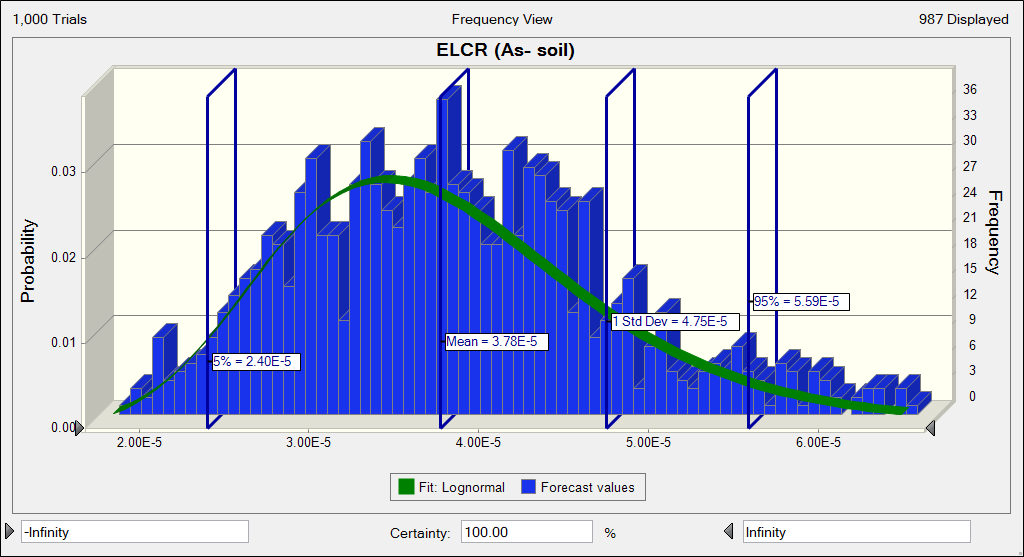


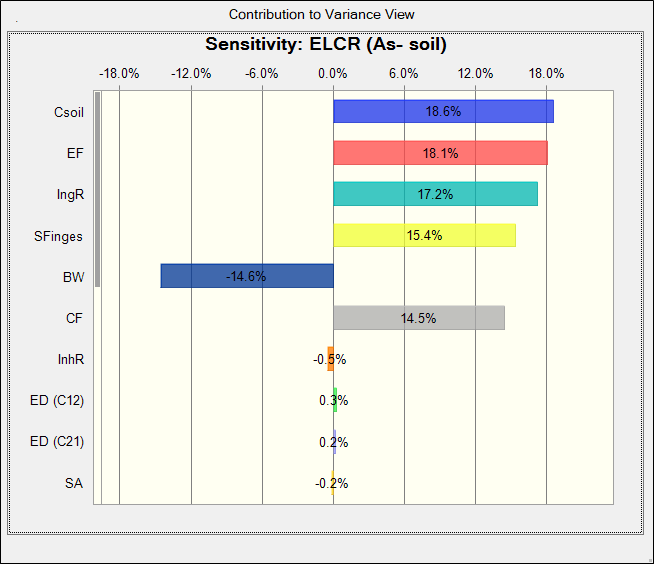


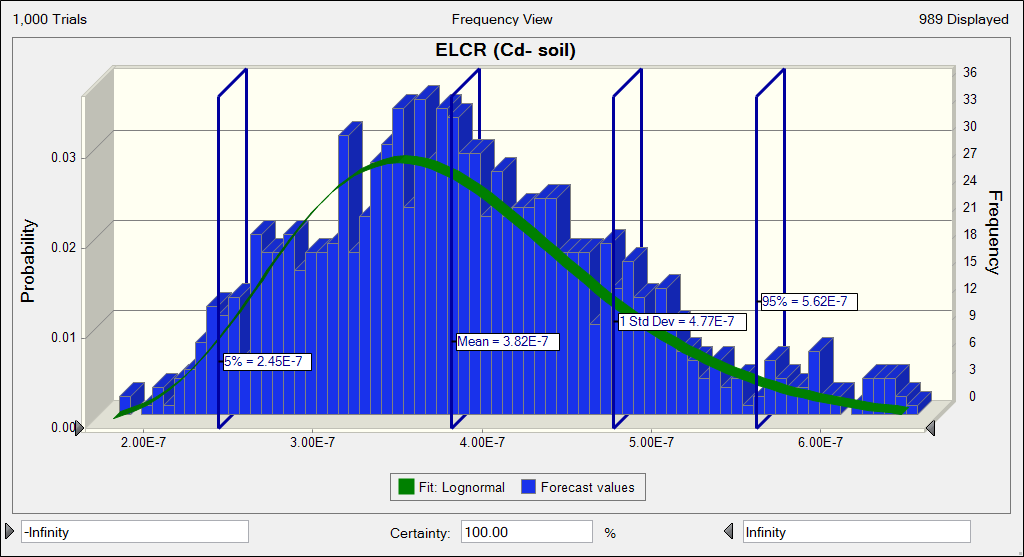


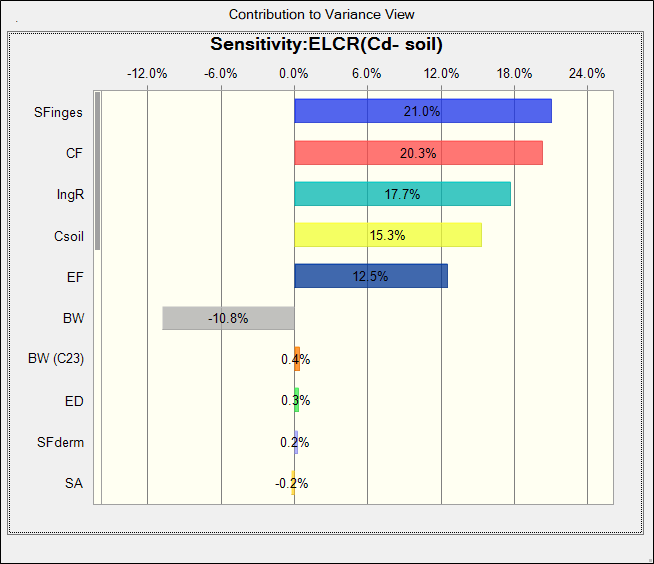


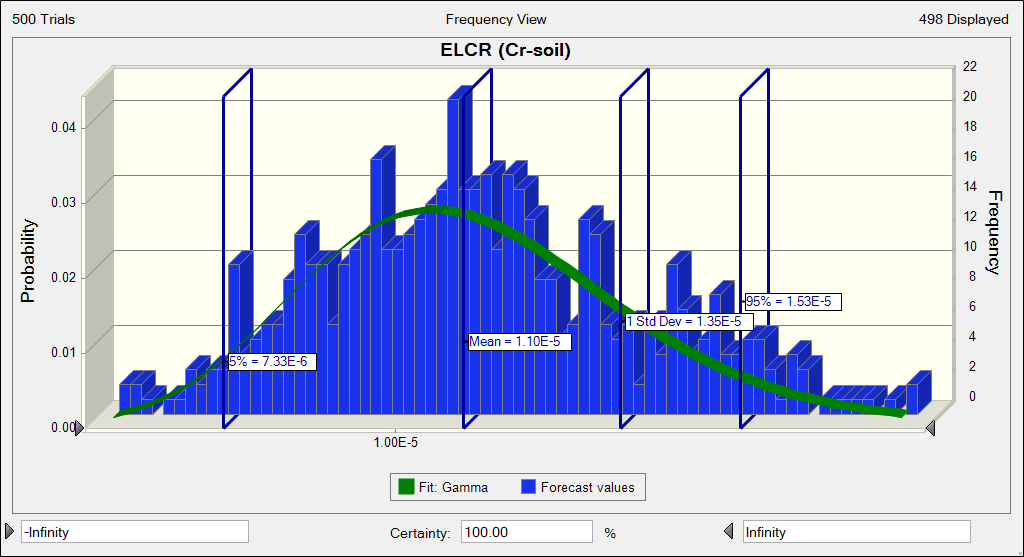


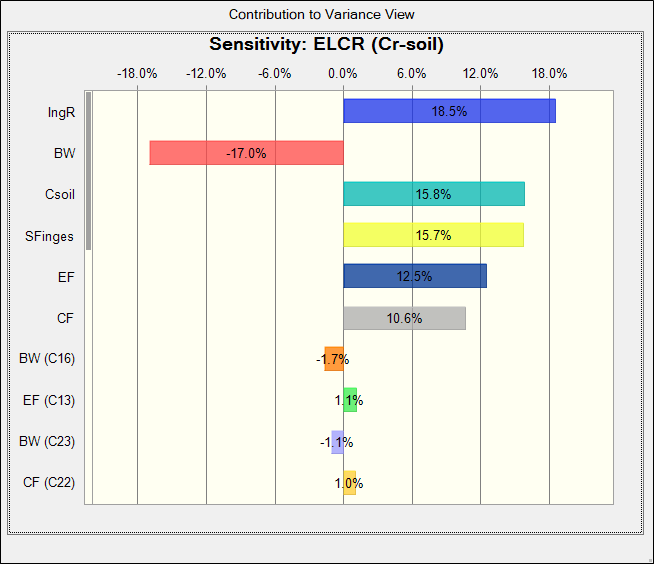


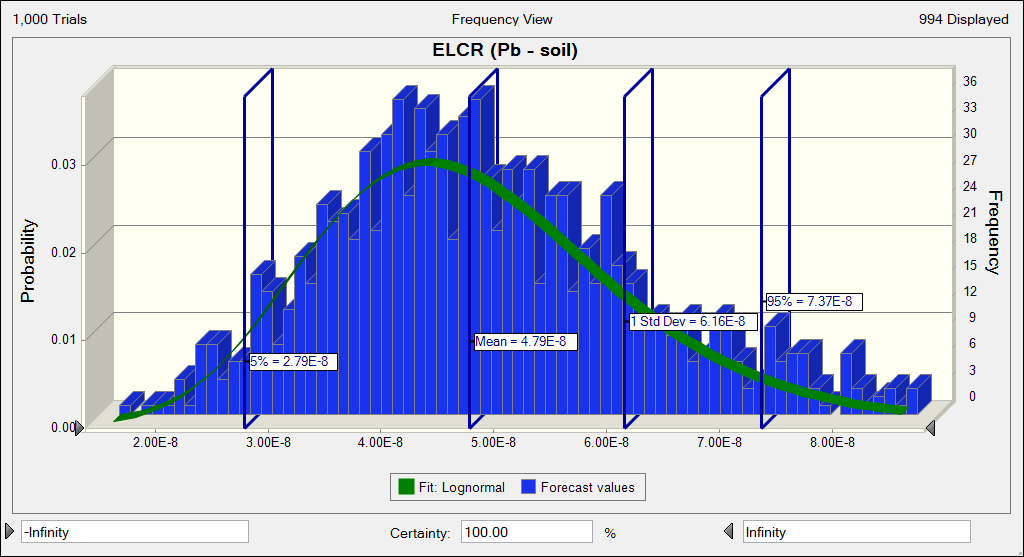


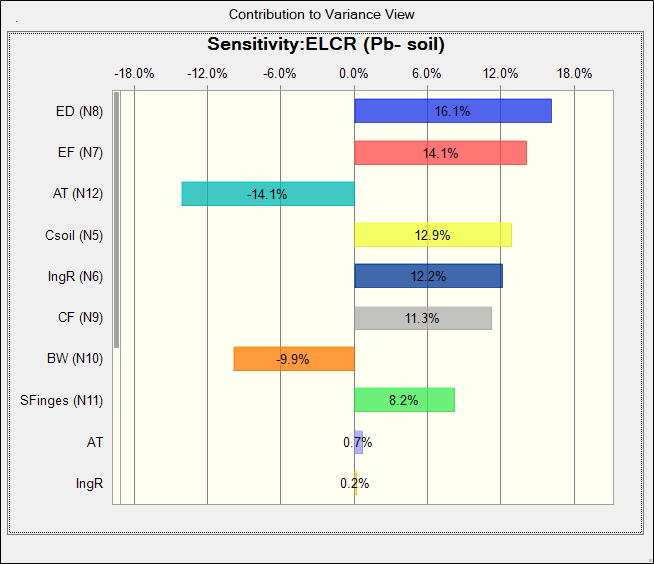


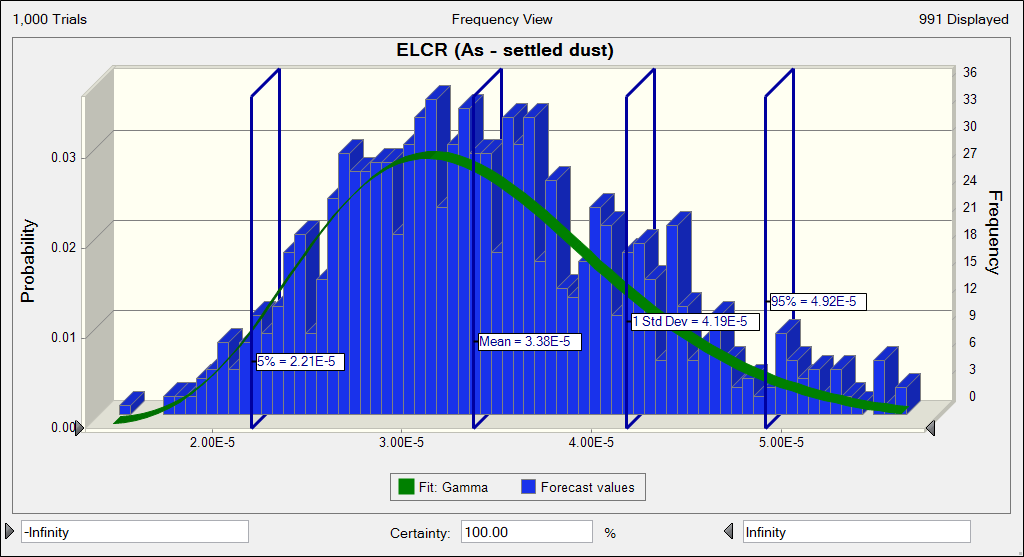


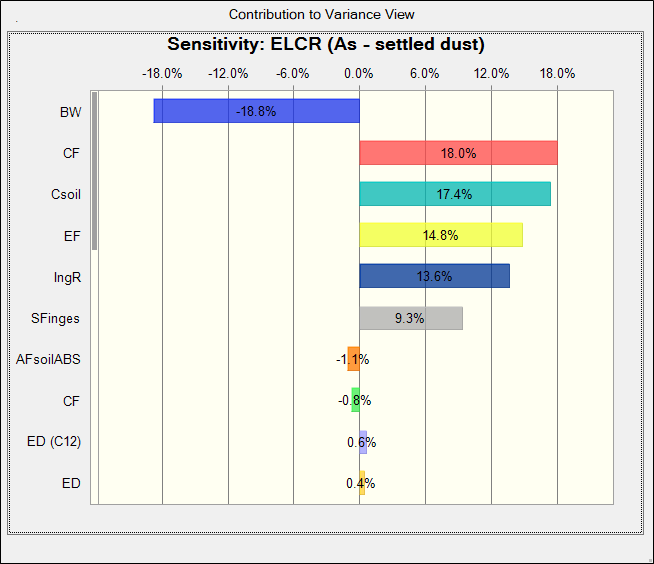


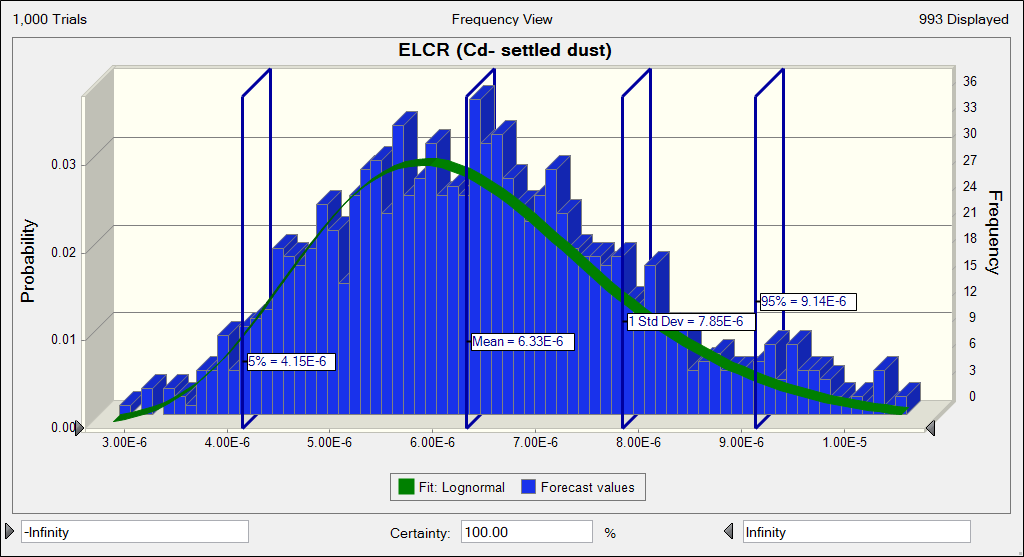


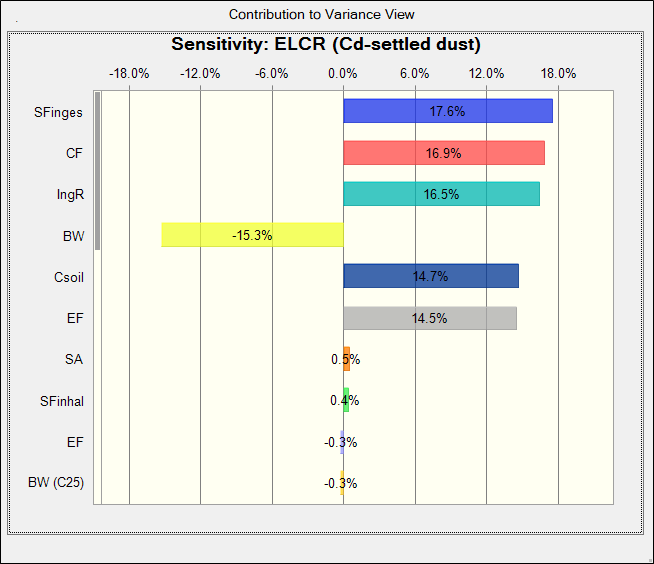


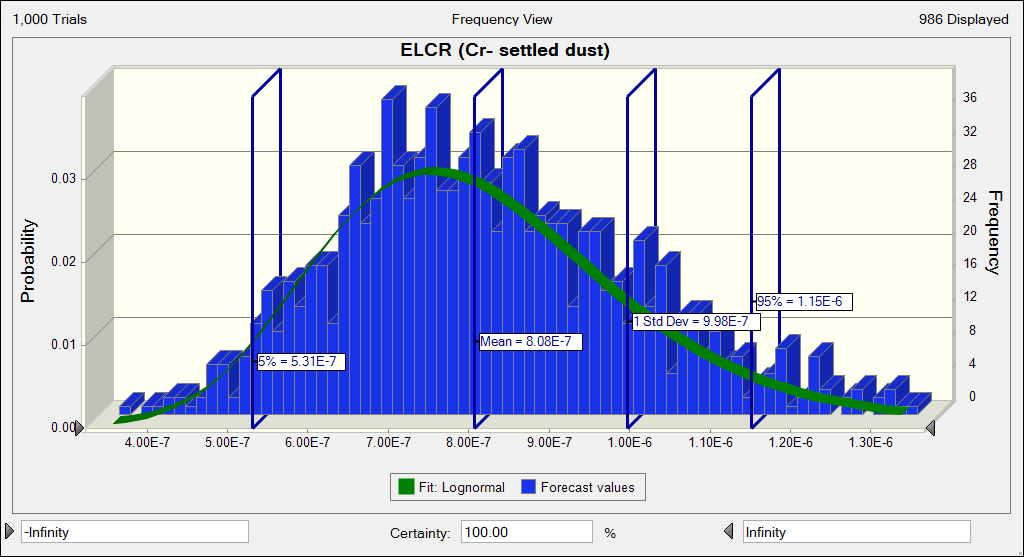


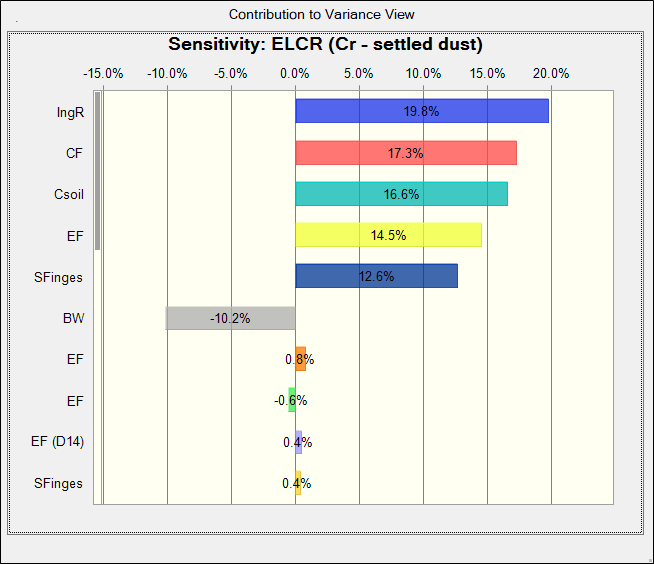


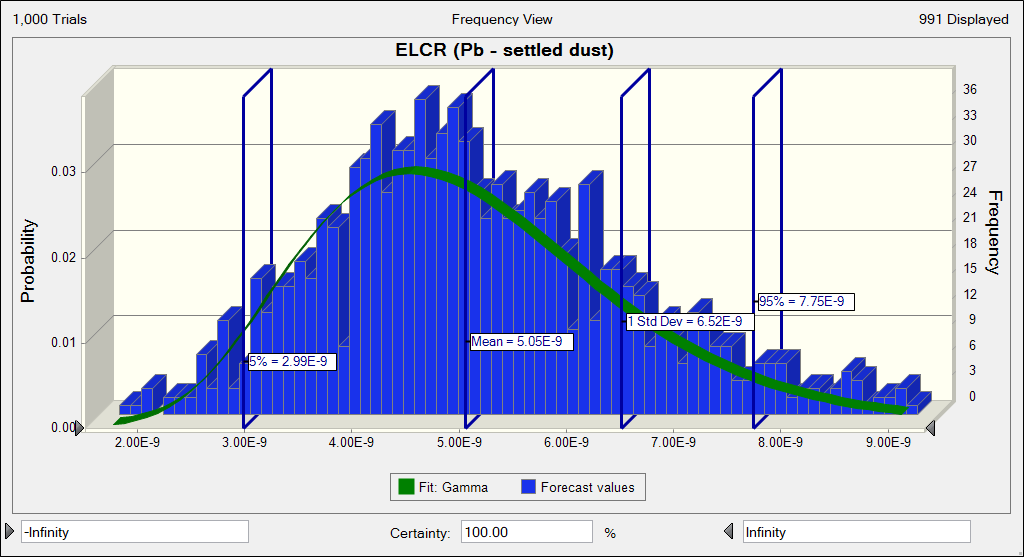


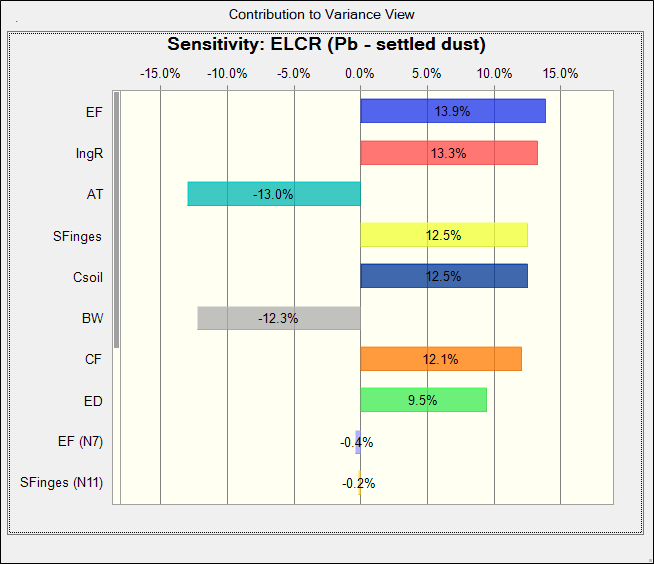


Figure S4 Simulation histogram for ELCR caused by HMs, and Sensitivity analyses
